# The Relief of Pain during Dental Operations

**Published:** 1910-04-15

**Authors:** George T. Gregg

**Affiliations:** Pittsburg, Pa.


### THE RELIEF OF PAIN DURING DENTAL OPERATIONS.

BY GEORGE T. GREGG, D.D.S., PITTSBURG, PA.

Since the beginning of my dental career I have tried all means at hand to relieve pain, and my efforts have repaid me many-fold, not only in gratitude from my patients but because I can do better work and shorten the sittings, thereby preventing strain on myself and saving my patients fatigue and shock. Patients leave the office, not in a state of dental fatigue, but generally happy in the thought that they can return and have the remainder of their work done without suffering. Patients whose teeth have been carried to the limit with gutta-percha and cement because they were not able to endure the agony of having the cavities prepared for permanent fillings, finding they are not to be hurt, will continue to come back until their mouths are in good condition; they are willing to allow me to undertake more extensive pieces of work. The old adage, "To do thorough work, you must hurt," should be reversed—"To do thorough work, you must not hurt." There is a very small percentage of patients who will undergo the pain of having a cavity thoroughly prepared along scientific lines.

Many inlays have failed from improper preparation, and not always because of the lack of knowledge. The operator, trying to avoid sensitive parts, depends too much on cement adhesion instead of on mechanical anchorage as well as cement.

*Gratitude.* If you wish your patients to speak well of you behind your back, don't hurt them. If you want to have them heap abuse upon you, torture them while they are in your chair. Is it not a pleasure to you to have a patient leave your chair saying: "Doctor, that was grand! you did not hurt me!"? It partly pays you for the operation. On the other hand, think of the patient who remarks, "You almost killed me!" and who, whenever your name is mentioned, has unkind things to say.

You can do a beautiful piece of work and find that it will not be appreciated so much as your efforts to alleviate pain. A filling at the gingival line on the buccal or labial surface will be pointed out with the remark, "My! oh my! how that hurts!" It may be a beautiful filling, but it is not the workmanship that is considered, but the suffering endured while the filling is being put in.

Dentistry at best is very trying, and a few words of gratitude once in a while make our lives happier. Every man who is inflicting pain undergoes severe strair, and in time that nervous strain will break down anyone, no matter how strong he may be. I have never been able to inflict pain without undergoing a strain. Many times I have lost sleep thinking of how I would excavate a buccal or labial cavity that could not be touched with an instrument without causing a wince. You all have had the same experience. There are patients whom you dread to see come into your office, because of the worry they cause you. These are cases in which we should be able to work without inflicting pain. They sap our nervous energy, put us in an irritable state, and send us home exhausted at the end of our day's work. If we can get through the day without such worries, we feel better, are happier, treat our patients better, and everything goes along smoothly.

### MEANS OF INDUCING ANESTHESIA.

The days of trying to desensitize cavities with hot air and topical applications of caustic soda, carbolic acid, formalin, cocain, sprays, and especially with sharp burs, are about over; at least they ought to be. These means are not practical and cannot be used with success. Sharp burs are good things to have for rapid cutting, but even with sharp burs you cannot excavate sensitive dentine without inflicting pain. They will alleviate pain but very little no matter how slowly you run your bur, or how delicate a touch you may have.

Cataphoresis has been tried, and found wanting. The rubber dam has always to be applied, and there are places where it cannot be used. Cataphoresis takes a great deal of time, the patient has to sit perfectly quiet, which is a great strain, and there is more or less pain connected with the application of the current; also you may cause the pulp to die at times, even after a half hour's application, the cavity will not be desensitized. Cavities below the gum line, along the buccal surfaces of upper and lower third molars, are simply out of the question with cataphoresis. The different sprays, such as ethyl chlorid and the Van Wyck-Kerr ether spray, will desensitize a cavity, but not without a good deal of time and patience, and with more or less pain in the majority of cases. The apparatus and the disagreable odor of the ether are very objectionable to the patient. I have made an effort to give a fair trial to anything new that I have heard of along this line, and have abandoned them all, except the high-pressure syringe and general anesthetics, especially nitrous ozid and oxygen—but never chloroform! To my mind, any one who administers chloroform or permits a physician to administer it in his chair is very foolish, and takes a great risk. Every month you can read of a death by chloroform that occurred in some dental office. Why should we administer chloroform, which shows a death-rate of about one in three thousand, when we can administer nitrous oxid and oxgyen, which has absolutely no death to its discredit?

Right here I want to recommend to you reading Dr. Wm. H. DeFord's new book on "General Anesthetics in Dentistry." No dentist who contemplates using a general anesthetic in his practice should be without it. He says: "Bold in other directions, commendably progressive in all that relates to manipulative ability and artistic development, the dental surgeon shrinks from anesthetics. He cuts living tissue, lacerating the nerves themselves, 'performing laparotomies upon the teeth,' so to speak, and the anesthetic usually employed is that of a witty speech or an amusing story, while the patient suffers, cringes, agonizes almost to the state of collapse. In this enlightened age no surgeon except a dental surgeon, permits a patient to undergo without an anesthetic, tortures equal in severity and duration to which one submits during the average dental operation."

Dr. Rhein recently reminded us that we as a profession occupy a unique position, inasmuch as we claim to have first introduced the benefits of general anesthesia by means of nitrous oxid, and yet to-day we find ourselves in the lamentable position of being subject to the criticism of the medical world because our work is so much dreaded and feared by patients. Dr. G. V. I. Brown said that if he should resume the general practice of dentistry, he would use nitrous oxid a thousand times where formerly he used it once.

### NITROUS OXID AND OXYGEN.

The majority of dentists are averse to administering general anesthetics. They say, "I don't extract teeth, and have no use for a general anesthetic." Extracting is a small part of our practice. I do not average one extraction in a month, yet probably administer an anesthetic, nitrous oxid and oxygen, every day in my practice, principally for the excavating of sensitive cavities. This has given me more satisfaction, afforded me more gratitude from patients, and helped me out of more tight places than anything else during my experience in dentistry. It will do just the same for everyone who spends a little time in learning how to administer it. Nitrous oxid and oxygen can be used in any painful operation that comes under the head of operative dentistry, such as excavating sensitive dentine, exposing congested pulps, lancing abscesses, desiccating cavities, amputating roots, and extracting teeth. The results are always positive. If you get your patient in the proper state, you can be sure of painlessly excavating cavities that would otherwise not bear the touch of cotton.

The value of this method consists in that it is universal. First get the confidence of your patient; this is absolutely essential for success. Every man has his own way of doing this. If your patients believe in you you will have little trouble. If a patient presents for the first visit, I have found it a good plan to study him and get a good idea of the temperament with which I have to deal, before adopting any manner of procedure. If the patient has had some unpleasant experience with an anesthetic, allay his fears. Tell him that the anesthetic which he is to take is nothing like ether or chloroform, that there will be no bad effects, that it is perfectly safe, that he will not become unconscious, and will feel perfectly normal a few moments afterward. Show him that you are sincere in trying to relieve him from pain, and you will have little trouble. Once a patient has taken nitrous oxid and oxygen for the excavation of a sensitive cavity, your troubles with that patient will be over.

Never mention laughing gas to your patients. Generally they have heard some exaggerated story of laughing gas, and you might excite them more. If they ask what gas it is, simply say it is nitrous oxid and oxygen.

See that your patient is comfortably seated and can breathe freely; take the ordinary precautions; adjust the nasal inhaler ,and tell him to inhale and exhale through the nose, till a peculiar feeling comes over him. Impress on him not to become alarmed at the odd feeling, that he must be in that state in order to get the proper effect. Have him inform you at the approach of the feeling and proceed to operate. If you cause pain, have the patient breathe in a little more; very seldom will you need to let him become unconscious to accomplish the desired result. Have your patient keep his eyes open, reassure him by talking to him, and you can operate in this state almost indefinitely. Of course you must have an assistant to operate the gases; you yourself must devote your entire attention to your patient.

I have been using this method with very few exceptions, for three years. In that time I have had only two cases that I would really call failures, and only in two cases nausea was experienced. One of these was a young man to whom I administered the anesthetic for the accommodation of a surgeon, and whom I kept in an unconscious state with nitrous oxid and oxygen for one hour and twenty minutes, using three cylinders of nitrous oxid and one of oxygen. The patient had not been prepared for an anesthetic. In both cases the sickness was of short duration. To-day I would be inclined to relinquish general practice rather than conduct it without the aid of nitrous oxid and oxygen.

A surgeon who had a serious operation to perform on a patient to whom he was afraid to administer any other anesthetic asked me to administer it for him in one of the hospitals in Pittsburg last July. I kept that patient under the influence of the anesthetic for about thirty-five minutes, for the removal of a cancerous growth around the kidney. The patient seemed perfectly rational before being removed from the operating table. Just a few minutes after the operation I spoke to him and asked him how he felt. The surgeon subsequently ordered one of these anesthetic apparatus, and just before I came away he performed two laparotomies, and was delighted with the result. Neither patient was nauseated, and both seemed perfectly comfortable.

One of the arguments against this method of anesthesia has been that you cannot get relaxation enough to operate on the abdomen; but this is not true. Dr. Teter has proved it, and the surgeon of whom I spoke came back to Pittsburg and performed these very operations.

### GREAT ADVANTAGE IN HEATING THE GAS.

It has been proved that by heating the gas you can reduce the amount required for anesthesia from one-third to one-half; moreover, the patient takes it much more easily. I use the Teter outfit, which I think is the most complete. Dr. Teter recently reported 13,000 operations with nitrous oxid and oxygen, which is very different from nitrous oxid alone. I think the heating was first suggested by Dr. DeFord, who has been administering nitrous oxid for about twenty-six years, and has been using it in the heated state for about three years. The results are wonderful; the anesthetic does not irritate the patient's throat, he is more quiet, and the mixture is much more effective. The apparatus has four cylinders, and there is a bag of nitrous oxid on one side, and one for oxygen on the other. The gas passes into the bags from the cylinders, and from there into a mixing chamber in the center; from this mixing chamber it is breathed by a tube passing into the nostril. This copper tube is immersed in water at 120 degrees, which is sufficient to warm it to the required degree.

The Teter inhaler, which I generally use, is not in the way, and you can go ahead with the operation without paying attention to the inhaler. When I wish to excavate a labial cavity, or if a patient has a short lip, or cannot breathe freely through the nostril, I use a Clark Correct Inhaler. This inhaler is not often needed, but when you want it, you want it badly. It is a wise precaution to have a large supply of these inhalers at hand, to suit different cases.

A young lady's teeth had been temporarily filled with gutta-percha. She was afraid to have them excavated. When she came to me she was very nervous, but I gained her confidence by taking out under cocain anesthesia a small root which she had in her mouth. I proved to her that I could do that without hurting. Then I told her I would excavate a couple of cavities with the aid of nitrous oxid and oxygen, and I did so without hurting her. After that, I had absolutely no trouble. She had two weeks in which to get ready for going to college, and I did a lot of work for her in that time.

For a young man, I inserted thirty odd fillings. He was a comical sort of fellow, and he said, "This is a very attractive way of accumulating a jag with no hang-over!"

In one family, I gave the gas to the father, who was over sixty years old, to the daughter, and to both sons.

One lady wanted to be absolutely unconscious before she was touched. On the back of her record she wrote a little note, in which she stated that it afforded her great pleasure to fill out the blank, and that she could not find words to express the relief that nitrous oxid and oxygen had given her.

### HINTS.

The high-pressure syringe is an instrument which, in my estimation, no dentist can afford to be without. There are many cases in which it can be used, especially in removing pulps in the preparation of abutments for bridge work. It will save a wonderful lot of time for you, and will practically eliminate the pain. I have used it a great deal in the preparation of cavities, but have found nitrous oxid and oxygen more practical for that purpose. It takes less time.

There are two items which I should like to emphasize, namely, the kind of pit to make and the kind of point to use. The first points which were gotten out were tapered so as to fit tightly in a hole drilled with a small round bur. Dr. Porter of Oil City, Pa., suggested to me the use of a needle without any taper, and a tapered hole in the tooth. This method has been a wonderful improvement over the straight-side hole and tapered needle. The square point cuts into the sides of the tapered hole and makes a perfectly tight joint, which makes it possible to apply much less force to keep contact. The point of the needle is the same as an ordinary hypodermic point, necessarily making the pit small.—*Cosmos*, March 1910.

